# Integrative Transcriptome Profiling Reveals *SKA3* as a Novel Prognostic Marker in Non-Muscle Invasive Bladder Cancer

**DOI:** 10.3390/cancers13184673

**Published:** 2021-09-17

**Authors:** Chaelin You, Xuan-Mei Piao, Keunsoo Kang, Yong-June Kim, Kyuho Kang

**Affiliations:** 1 Department of Biological Sciences and Biotechnology, Chungbuk National University, Cheongju 28644, Korea; clyu104@cbnu.ac.kr; 2 Department of Urology, College of Medicine, Chungbuk National University, Cheongju 28644, Korea; phm1013@hotmail.com; 3 Department of Microbiology, College of Science & Technology, Dankook University, Cheonan 31116, Korea; kangk1204@dankook.ac.kr

**Keywords:** non-muscle invasive bladder cancer, prognostic marker, *SKA3*, survival, transcriptomics, tumor progression

## Abstract

**Simple Summary:**

Approximately 80% of all new bladder cancer patients are diagnosed with non-muscle invasive bladder cancer. However, about 15% of them progress to muscle-invasive bladder cancer, which is associated with poor prognosis. As these diseases are highly heterogeneous, the identification of an accurate prognostic biomarker for them has been challenging. In the present study, our aims were to compare cancerous and normal adjacent bladder tissues excised from patients in various cohorts and endeavor to detect differentially expressed genes known to be upregulated exclusively in patients at high risk of bladder cancer progression. Through various molecular methods, we found that the expression level of the cell cycle-regulating *SKA3* gene was markedly increased in patients with worse prognosis and high progression risk. Hence, *SKA3* is a viable predictive marker for bladder cancer progression and its inhibition could constitute part of a novel therapy that improves the prognosis of this disease.

**Abstract:**

Approximately 80% of all new bladder cancer patients are diagnosed with non-muscle invasive bladder cancer (NMIBC). However, approximately 15% of them progress to muscle-invasive bladder cancer (MIBC), for which prognosis is poor. The current study aimed to improve diagnostic accuracy associated with clinical outcomes in NMIBC patients. Nevertheless, it has been challenging to identify molecular biomarkers that accurately predict MIBC progression because this disease is complex and heterogeneous. Through integrative transcriptome profiling, we showed that high *SKA3* expression is associated with poor clinical outcomes and MIBC progression. We performed RNA sequencing on human tumor tissues to identify candidate biomarkers in NMIBC. We then selected genes with prognostic significance by analyzing public datasets from multiple cohorts of bladder cancer patients. We found that *SKA3* was associated with NMIBC pathophysiology and poor survival. We analyzed public single-cell RNA-sequencing (scRNA-seq) data for bladder cancer to dissect transcriptional tumor heterogeneity. *SKA3* was expressed in an epithelial cell subpopulation expressing genes regulating the cell cycle. Knockdown experiments confirmed that *SKA3* promotes bladder cancer cell proliferation by accelerating G2/M transition. Hence, *SKA3* is a new prognostic marker for predicting NMIBC progression. Its inhibition could form part of a novel treatment lowering the probability of bladder cancer progression.

## 1. Introduction

Bladder cancer is the most prevalent cancer of the urinary tract and has the thirteenth highest cancer mortality rate worldwide [1]. Moreover, its prognosis and survival have not improved over the past few decades [2,3]. Bladder cancer is classified either as non-muscle invasive bladder cancer (Ta–T1; NMIBC) or muscle-invasive bladder cancer (T2–T4; MIBC), depending on whether the tumor has invaded the muscle layer [4,5]. Approximately 75–80% of all newly diagnosed bladder cancer patients are NMIBC while 20–25% are MIBC [6,7,8]. Despite transurethral resection (TUR) and intravesical chemotherapy, over 40% of all NMIBC tumors recur and approximately 10% of all NMIBC-diagnosed patients progress to MIBC [9,10]. It is difficult to identify the latter as its molecular characteristics are highly variable during progression [11,12]. Moreover, MIBC patients are relatively more likely to develop metastasis than NMIBC patients and the survival rates are significantly lower in the former than the latter [13,14]. Hence, early cystectomy is sometimes considered for patients with aggressive NMIBC in order to prevent progression to MIBC [4,15]. There is an urgent need for reliable, high-specificity biomarkers that can accurately predict prognoses and clinical outcomes in bladder cancer patients.

Bladder cancer prognosis is determined primarily on the basis of the pathological stage of the tumor [16]. Urine cytology is a standard test in bladder cancer diagnosis but it has low overall sensitivity (<40%) [17]. To overcome these limitations, investigations have been conducted to find biomarkers based on cancer tissue gene expression profiles. Certain mRNA markers accurately predict cancer prognosis [18,19]. Transcriptome profiling using cancer tissue samples could lead to the discovery of precise molecular markers for cancer diagnosis and prognosis [20]. Several studies showed that *CCNB1, FOXM1, GSN*, *LAMC2*, and other genes may serve as prognostic gene expression markers for NMIBC [21,22,23,24]. Simultaneous evaluation of multiple biomarkers in bladder cancer has increased as these tumors are complex and heterogeneous [25]. However, few molecular biomarkers that accurately predict NMIBC prognosis have been identified.

Spindle and kinetochore associated complex subunit 3 (*SKA3*) is a member of the SKA complex. In humans, it is an essential mitotic component required for accurate cell division. *SKA3* coordinates checkpoint signaling at microtubule binding sites within kinetochores. In this manner, it silences spindle checkpoints when the chromosomes are aligned at metaphase, thereby allowing timely initiation of anaphase and termination of mitosis [26,27]. Previous studies showed that *SKA3* plays important roles in the pathogenesis and exacerbation of hepatocellular carcinoma (HCC), cervical cancer, and other malignancies. A recent study reported that *SKA3* participates in HCC through p53 signaling and promotes cancer progression by inhibiting CDK2/p53 phosphorylation [28]. In cervical cancer, *SKA3* activates PI3K-Akt signaling, regulates cell cycle progression, and exacerbates tumors [29]. However, the roles of *SKA3* in bladder cancer have not been elucidated.

An objective of the present study was to identify prognostic markers for NMIBC patients at a high risk for progression. By performing RNA-seq on both bladder tumor and normal adjacent tissues (NAT) in NMIBC patients, we identified significantly upregulated genes. *SKA3* was strongly correlated with the pathology and poor clinical outcomes of NMIBC. Furthermore, scRNA-seq analysis of bladder cancer revealed that *SKA3* was upregulated in certain epithelial cell subpopulations with a high probability of proliferation. Our data also showed that *SKA3* promotes bladder cancer cell proliferation by accelerating G2/M transition. The foregoing results demonstrate that *SKA3* might be a novel diagnostic and prognostic marker for bladder cancer patients.

## 2. Materials and Methods

### 2.1. Patients and Tissue Samples

Table 1 shows the baseline characteristics of the case subjects (*n* = 24 bladder tissue samples) for RNA sequencing. Sixteen samples were excised from NMIBC patients and the eight others originated from NAT comprising a control set. Table 2 shows the baseline characteristics of the case subjects (*n* = 105 bladder tissue samples) for validation. Of these, 89 were obtained from NMIBC patients and histologically verified as transitional cell carcinomas while the remaining 16 consisted of normal bladder mucosa samples and served as the control set. To reduce the risk that confounding factors could affect the analyses, patients diagnosed with concomitant in situ carcinoma or in situ carcinoma lesions alone were excluded. Fresh frozen specimens were acquired from surgical transitional cell carcinoma resections at CBNUH. All tumors were macro-dissected within 15 min of surgical resection. Each specimen was confirmed by pathological analysis of a portion of the fresh frozen specimens obtained by TURBT. Tumors were staged according to the 2017 TNM Classification and graded according to the 2004 WHO Classification based on standard criteria [30]. Each patient was followed and managed per standard recommendations [31,32,33]. Surveillance was performed by cystoscopic examination and upper urinary tract imaging according to the guidelines of the European Association of Urology [32]. Recurrence was defined as a relapse of primary NMIBC of the same pathological stage. NMIBC and MIBC progression were defined as TNM stage progression after disease recurrence. The mean follow-up period was 70.12 mo (range, 10.70–174.90 mo) for NMIBC patients.

### 2.2. RNA Sequencing Analysis

Total RNA was extracted from all tissues with TRIzol reagent (Invitrogen, Carlsbad, CA, USA) as previously described [34] and stored at −80 °C. RNA content was measured with Quant-IT RiboGreen (Invitrogen). One hundred nanograms of total RNA was used to construct the sequencing library in an Illumina NovaSeq 6000 system (Illumina, San Diego, CA, USA) in 2 × 100-bp paired-end mode.

The FASTQ files generated by sequencing were quality-processed, and adapter sequences were removed with TrimGalore. Processed raw reads were mapped to the human genome (*hg38*) with STAR using the default parameters [35]. HOMER was used to convert the aligned reads into tag directories [36]. Each file was quantified with the “analyzeRepeats” script in HOMER. Gene expression levels in each sample were normalized by fragments per kilobase of transcript per million mapped reads (FPKM). DEGs were identified by DESeq2 analysis using the raw read counts [37]. The “getDifferentialExpression” command in HOMER was used at adjusted *p*-value < 0.05, fold change > 2, and FPKM > 2. A gene ontology (GO) analysis was performed with Metascape [38]. A gene set enrichment analysis (GSEA) was conducted to identify any DEGs enriched in the gene lists extracted from MSigDB [39] and determine enrichment in the gene sets from the Hallmarks collection.

### 2.3. Public Expression Profiles

The expression profiles of GSE13507 (67 normal, 103 NMIBC, and 62 MIBC samples), GSE121711 (10 normal, five NMIBC, and four MIBC samples), and GSE32548 (92 NMIBC and 39 filtered MIBC samples) were downloaded from GEO. E-MTAB-4321 (450 NMIBC samples, 10 NMIBC samples filtered with CIS or PULNMP, and 16 filtered MIBC samples) was downloaded from EMBL-EBI. Gene expression RNA-seq (HTSeq-FPKM GDC Hub) and the corresponding clinical characteristics of the Bladder Cancer (BLCA) cohort in The Cancer Genome Atlas (TCGA) (19 normal and 411 MIBC samples) were downloaded from the UCSC Xena database and used as a test set for external validation.

### 2.4. DEG Analysis

A two-tailed *t*-test was used to identify DEGs of T1 vs. Ta and high-grade vs. low-grade in tissues from NMIBC patients in the E-MTAB-4321 cohort. This statistical test compares mean expression levels between two experimental groups and evaluates the significance of the difference relative to the variance of the data within groups. Fold change was computed as the ratio of gene expression levels in two experimental groups. The Gene Expression Profiling Interactive Analysis 2 (GEPIA2) database [40] was used to detect DEGs between bladder cancer and normal (non-tumor) bladder tissues.

### 2.5. Single-Cell RNA-Seq Dataset Analysis of Public Data

Previously published scRNA-seq data from CD45-negative cells in human bladder cancer tissues [41] were used to identify cell populations with relatively upregulated SKA3. Only CD45-negative cells with mitochondrial RNA comprising <20% of the total were included. A uniform manifold approximation and projection (UMAP) of 3774 CD45-negative cells obtained from two patient samples was plotted with PCA and clustered with the Seurat v. 3.1.5 package in R v. 3.6.2 (R Core Team, Vienna, Austria). Cells in the scRNA-seq dataset were classified via marker genes into the following lineages: epithelial cells (marked with *KRT19* and *EPCAM*), fibroblasts (marked with *SFRP2*), endothelial cells (marked with *PLVAP*) and myofibroblasts (marked with *RGS5*). The epithelial compartment was then classified into Epithelial cells-1, Epithelial cells-2, and Epithelial cells-3 based on their regulatory pathways.

### 2.6. Cell Culture

Human bladder cancer cell lines 5637 and T24 were cultured in RPMI1640 (No. 11875-119; Gibco, Grand Island, NY, USA) supplemented with 10% fetal bovine serum (FBS; No. 16000-044; Gibco) and 1% penicillin-streptomycin (No. 15070-063; Gibco). All cell lines were routinely cultured at 37 °C in a humidified 5% CO_2_ incubator.

### 2.7. RNA Interference

Human *SKA3* gene was knocked down with SMART pool ON-TARGETplus siRNA (No. L-015700-00-0005; Dharmacon Inc., Lafayette, CO, USA). ON-TARGETplus Non-targeting Pool was used as a negative control (No. D-001810-10-05; Dharmacon Inc.). T24 and 5637 cells were seeded into a six-well or 24-well plate, incubated for 24 h, and transfected with RNA duplex and Lipofectamine^TM^ RNAi-MAX (Invitrogen) in Opti-MEM I reduced serum medium (Thermo Fisher Scientific, Waltham, MA, USA) without antibiotics according to the manufacturer’s instructions. After 24 h, the medium was replaced with complete medium including antibiotics and the cells were harvested for the subsequent experiments after 24 h at 37 °C in a humidified 5% CO_2_ incubator.

### 2.8. RNA Extraction and Real-Time Quantitative PCR

Total RNA was extracted with Ribospin II (GeneAll Biotechnology, Seoul, Korea), and 0.5 µg total RNA was reverse-transcribed with a RevertAid First Strand cDNA Synthesis kit (Thermo Fisher Scientific). Real-time qPCR was performed with TOPreal qPCR 2X PreMIX (SYBR Green with low ROX; No. RT500M; Enzynomics Co. Ltd., Daejeon, Korea). The following primers were used to amplify the candidate *SKA3* gene: (Gene ID No. 221150): sense, 5′-GAAGTATGGATATAGTCCACG-3′ (21 bp; T_m_ = 58 °C); antisense, 5′-GACTACGTGGAGACTTCTCAG-3′ (21 bp; T_m_ = 64 °C). The amplicon size was 152 bp. Gene expression was normalized to that of TBP or GAPDH. The following primers were used to amplify TBP (Gene ID No. 6908): sense, 5′-CCCGAAACGCCGAATATAATCC-3′ (22 bp; T_m_ = 61.2 °C); antisense, 5′-AATCAGTGCCGTGGTTCGTG-3′ (20 bp; T_m_ = 63 °C). The amplicon size was 80 bp. The following primers were used to amplify GAPDH (Gene ID No. 2597): sense, 5′-CATGTTCGTCATGGGTGTGA-3′ (20 bp; T_m_ = 60 °C); antisense, 5′-ATGGCATGGACTGTGGTCAT-3′ (20 bp; T_m_ = 60 °C). The amplicon size was 156 bp. The real-time qPCR conditions were as follows: one cycle at 95 °C for 10 min followed by 50 cycles of 10 s at 95 °C for denaturation, 15 s at 60 °C for annealing, and 20 s at 72 °C for extension. The melting program was performed at 72–95 °C and a heating rate of 1 °C/45 s. Rotor-Gene Q v. 2.3.1 (Qiagen, Hilden, Germany) was used to capture and analyze the spectral data. All experiments were repeated in triplicate.

### 2.9. Colony Formation Assay

Transfected bladder cancer cells were plated in triplicate in six-well plates at a density of 1000/well. After culturing for 7 days, the colonies were washed, fixed in methanol for 10 min, and stained with 0.5% (*w*/*v*) crystal violet solution for 15 min. Cell confluency per well was quantified using the “colony area” plugin of ImageJ [42]. All experiments were repeated in triplicate.

### 2.10. Flow Cytometry

The 5637 and T24 cell lines were transfected with non-targeting control and *SKA3* siRNAs. After 48 h, the cells were harvested with TrypLE Express (No. 12604013; Gibco).

To detect intracellular *SKA3*, the cells were fixed and permeabilized in Cyto-Fast^TM^ Fix/Perm buffer solution (No. 426803; BioLegend, San Diego, CA, USA) at room temperature for 20 min. The cells were then washed and labeled with primary *SKA3* antibody (No. A304-215A; Bethyl Laboratories Inc., Montgomery, TX, USA) or primary purified rabbit polyclonal isotype ctrl antibody (No. 910801; BioLegend) diluted with cell-staining buffer (No. 420201; BioLegend) on ice for 1 h. The cells were subsequently stained with PE-conjugated donkey anti-rabbit IgG (minimal x-reactivity) secondary antibody (No. 406421; BioLegend) at room temperature for 20 min. The stained cells were analyzed on a CytoFLEX (Beckman Coulter, Brea, CA, USA) and the files were plotted with FlowJo v. 10.7 (FlowJo LLC, Ashland, CO, USA).

To detect the cell cycle, the cells were fixed in ice-cold 70% (*v*/*v*) ethanol with phosphate-buffered saline (PBS) and stained with FxCycle propidium iodide (PI)/RNase staining solution (No. F10797; Invitrogen). The DNA content was evaluated by flow cytometry with a CytoFLEX (Beckman Coulter). Data were analyzed with Kaluza software v. 2.1 (Beckman Coulter). All experiments were repeated in triplicate.

### 2.11. Statistical Analysis

Univariate Cox proportional hazard regression models were used to assess the prognostic value of the candidate genes for RFS and PFS in the GSE13507 cohort. The hazard ratios (HRs) and 95% confidence intervals (CIs) were calculated to determine relative risk. The gene expression values were log2-transformed and median-centered across all samples in the CBNUH validation cohort. The significance of various clinicopathological variables was evaluated with univariate and multivariate Cox proportional hazard regression models. Survival curves for the GSE13507 and E-MTAB-4321 cohorts were plotted by the Kaplan–Meier method to determine the prognostic value of the genetic biomarker. They were then compared by the log-rank test. *p* < 0.05 was considered statistically significant. Patient samples in each dataset were divided into high- and low-expression groups based on the median expression value of each gene. GEPIA2 was used to generate disease-free survival (DFS) curves based on gene expression and the log-rank test for bladder cancer.

Statistical tests were selected on the basis of appropriate assumptions for data distribution and variance characteristics. Welch’s *t*-test was used on two unpaired samples. A Mann–Whitney *U* test was used to compare the *SKA3* expression levels in NMIBC and control tissues. Error bars indicated standard error of the mean (SEM). Statistical analyses were performed in IBM SPSS Statistics v. 20.0 (IBM Corp., Armonk, NY, USA). Graphs were plotted with GraphPad Prism v. 8 (GraphPad Software, La Jolla, CA, USA). *p* < 0.05 was considered statistically significant.

## 3. Results

### 3.1. Transcriptome Profiles Showing Molecular Signatures in Patients with NMIBC

**Figure 1:**
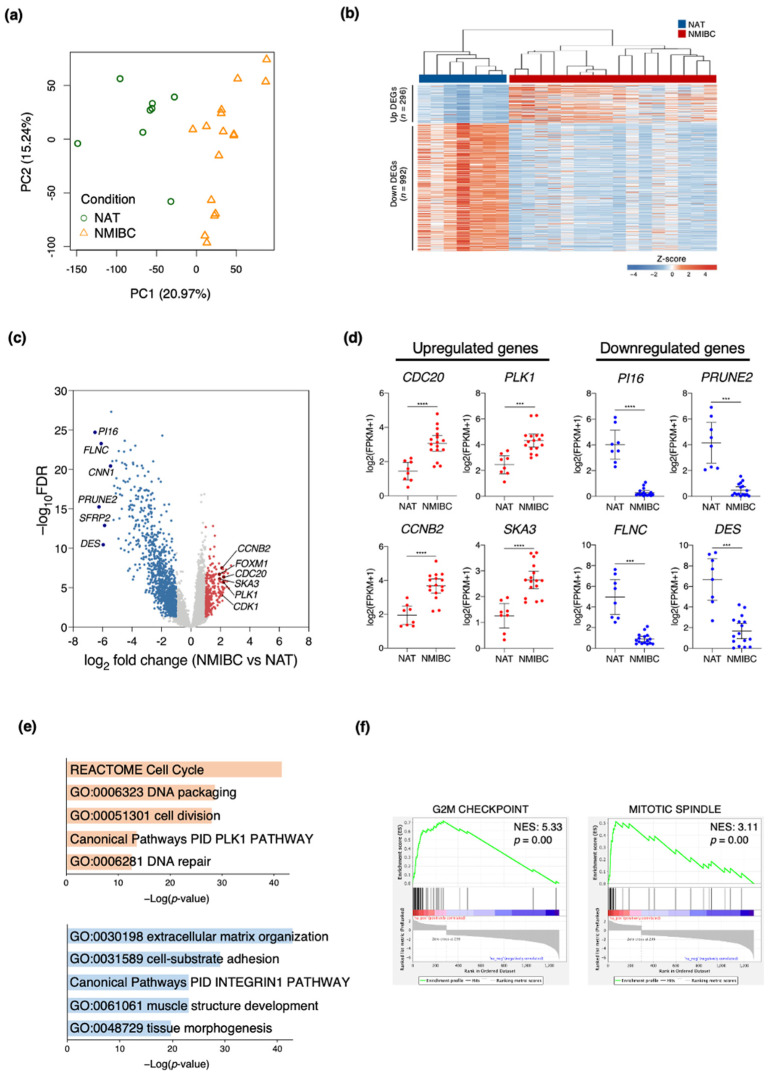
Analysis of gene expression in non-muscle invasive bladder cancer (NMIBC). (**a**) Principal component analysis (PCA) plot of 15,585 genes expressed in NMIBC (*n* = 16) and normal adjacent tissue (NAT) (*n* = 8) samples. (**b**) Hierarchical and *k*-means (*k* = 2) clustering of 1288 DEGs between NMIBC and NAT. Values are z-scores. (**c**) Volcano plot of RNA-seq data displaying transcriptomic changes between NMIBC and NAT. Colored dots correspond to genes with FDR < 0.05, > two-fold expression change, and FPKM > 2. (**d**) Expression levels of selected DEGs in NMIBC and NAT. Data are means ± SEM. *P* values were determined by Welch’s *t*-test. *** *p* < 0.001, **** *p* < 0.0001. (**e**) Gene ontology (GO) analysis of DEGs between NMIBC and NAT (red, upregulated; blue, downregulated). Important GO terms were selected based on FDR < 1.0 × 10^−10^. (**f**) Representative gene set enrichment analysis (GSEA) results of upregulated DEGs between NMIBC and NAT.

Several sets of NMIBC biomarkers have been proposed [21,22,23,24]. However, no reliable prognostic markers have been established. Hence, we performed RNA sequencing (RNA-seq) to profile the relative changes in gene expression in bladder tumor tissues (n = 16) and NAT (*n =* 8) excised from NMIBC patients. We then attempted to identify a set of prognostic biomarkers for NMIBC patients. The transcriptome profiles distinguished tumor tissues from NAT based on a principal component analysis (PCA) plot analyzed using the expression levels of all known genes (Table 1; Figure 1a). A comparison of the transcriptomes between the tumor tissues and NAT identified 1288 differentially expressed genes (DEGs) of which 296 were upregulated and 992 were downregulated (FDR < 0.05; >two-fold difference in expression level; FPKM > 2) (Figure 1b). Volcano and scatter plots showed that the gene expression levels in NMIBC significantly differed from those in NAT (Figure 1c). *CDC20*, *CCNB2*, *PLK1*, and *SKA3* were significantly upregulated whereas *PI16*, *PRUNE2*, *FLNC*, and *DES* were significantly downregulated in NMIBC (Figure 1d). To infer the potential functions of the DEGs, we conducted a gene ontology (GO) analysis in Metascape. The cell cycle (*p* = 2.98 × 10^−42^), cell division (*p* = 1.04 × 10^−28^), and DNA repair (*p* = 2.20 × 10^−13^) pathways were significantly enriched in NMIBC compared to NAT. All these pathways are associated with the cellular alteration characteristic of bladder cancer. By contrast, the extracellular matrix organization (*p* = 1.12 × 10^−43^) and muscle structure development (*p* = 8.93 × 10^−24^) pathways were significantly associated with the downregulated genes in NMIBC (Figure 1e). A gene set enrichment analysis (GSEA) showed that cell cycle-related pathways such as G2M checkpoint and mitotic spindle were significantly associated with the upregulated genes in NMIBC (Figure 1f). Overall, the results indicate that the cell cycle-related gene sets were significantly altered in NMIBC tumor tissues.

### 3.2. Identification of Genes Associated with NMIBC Prognosis

**Figure 2:**
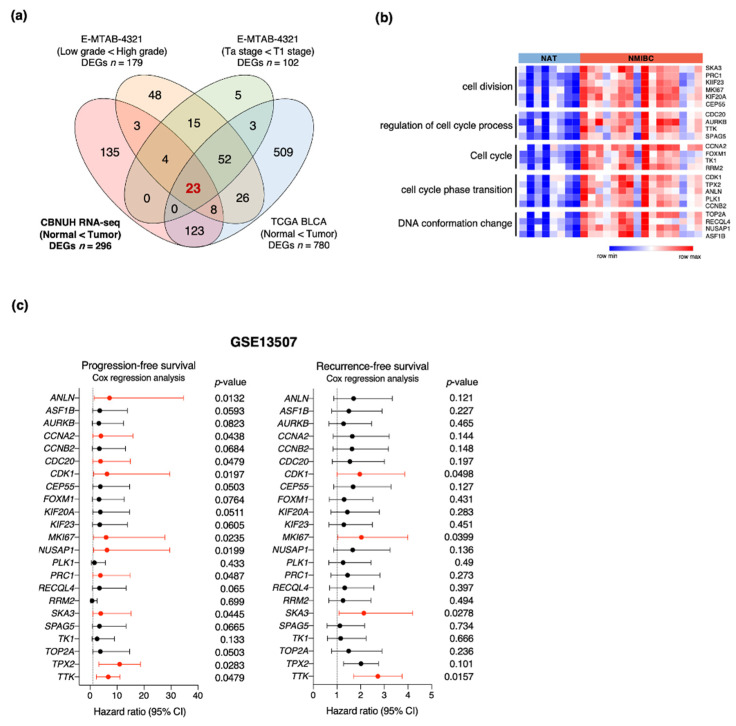
Identification of prognostic markers for NMIBC patients. (**a**) Venn diagram reveals intersections between DEGs in four categories including upregulated DEGs of CBNUH RNA-seq data and three categories from multiple cohorts (DEGs of T1 vs. Ta stage in E-MTAB-4321 cohort; DEGs of high-grade vs. low-grade tumor in E-MTAB-4321 cohort; and DEGs of bladder cancer vs. normal bladder in TCGA cohort; *p* < 0.001; fold change >1.7; average expression level >2. (**b**) Heatmap and GO term enrichment for 23 candidate genes. (**c**) Univariate analyses of progression-free survival (PFS) and recurrence-free survival (RFS) derived from Cox regression model. CI, confidence interval.

To identify reliable prognostic biomarkers for bladder cancer patients, we reanalyzed available RNA-seq data from two independent cohorts, namely, The Cancer Genome Atlas urothelial bladder carcinoma (TCGA-BLCA) and E-MTAB-4321 (UROMOL) [43]. We also used our own RNA-seq data designated CBNUH. The DEGs were identified in each cohort using the same criteria as those for the analysis of the CBNUH RNA-seq data. As the E-MTAB-4321 cohort contains tumor grade and staging information, we identified prognosis-related DEGs either by comparing high vs. low grades or by comparing T1 vs. Ta stages. All DEGs in these cohorts were then intersected to select the commonly upregulated prognosis-related genes in bladder cancers relative to their corresponding controls. We finally identified 23 genes (Figure 2a). A GO analysis showed that all of them were significantly associated with cell cycle-related pathways (Figure 2b). We analyzed an independent NMIBC cohort (GSE13507) with a Cox proportional regression model to estimate the prognostic significance of these genes (Figure 2c). Ten of the 23 genes were significantly associated with progression-free survival (PFS), while four (*SKA3*, *MKI67*, *CDK1*, and *TTK*) were related to recurrence-free survival (RFS) in NMIBC. The four latter genes were associated with worse clinical outcomes. We selected the *SKA3* gene for further analysis as its prognostic potential in bladder cancer patients has never been investigated.

### 3.3. SKA3 Expression Is Correlated with Clinicopathological Characteristics of Bladder Cancer

**Figure 3:**
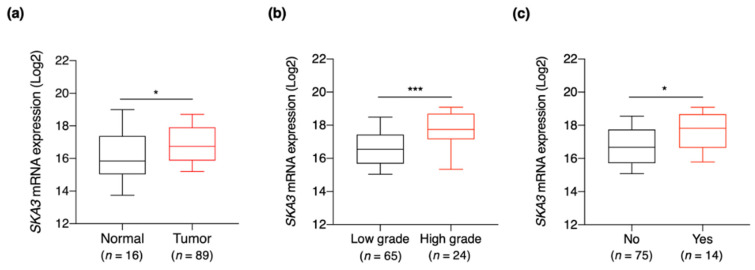
*SKA3* expression in NMIBC tissues. (**a**) *SKA3* expression was higher in NMIBC tissues than it was in control tissues. (**b**) *SKA3* mRNA expression was significantly upregulated in high-grade NMIBC tissues compared with low-grade NMIBC tissues. (**c**) *SKA3* mRNA expression was significantly higher in NMIBC patients who later progressed to MIBC than it was in patients who did not progress to MIBC. NMIBC, non-muscle invasive bladder cancer; MIBC, muscle-invasive bladder cancer. Control samples: normal bladder mucosae. Data are reported as 10th–90th percentiles. *p*-value determined by Mann–Whitney *U* test. * *p* < 0.05, *** *p* < 0.001.

To determine whether *SKA3* expression is a potential prognostic biomarker for NMIBC patients, we measured it by qPCR in tumor tissues from 89 NMIBC patients and in 16 normal bladder tissues (Table 2). The results of the RNA-seq data analysis (Figure 1 and Figure 2) showed that *SKA3* expression was significantly higher in NMIBC tissues than normal bladder mucosa (*p* < 0.05) (Figure 3a). Additionally, *SKA3* was significantly overexpressed in high-grade NMIBC tissues compared with low-grade NMIBC tissues (*p* < 0.001; Figure 3b). However, *SKA3* was significantly upregulated in the tumor tissues of NMIBC patients who had progressed to MIBC compared to those without progression (*p* < 0.05; Figure 3c). These results demonstrate that *SKA3* upregulation potentially serves as a prognostic biomarker for NMIBC patients.

**Figure 4:**
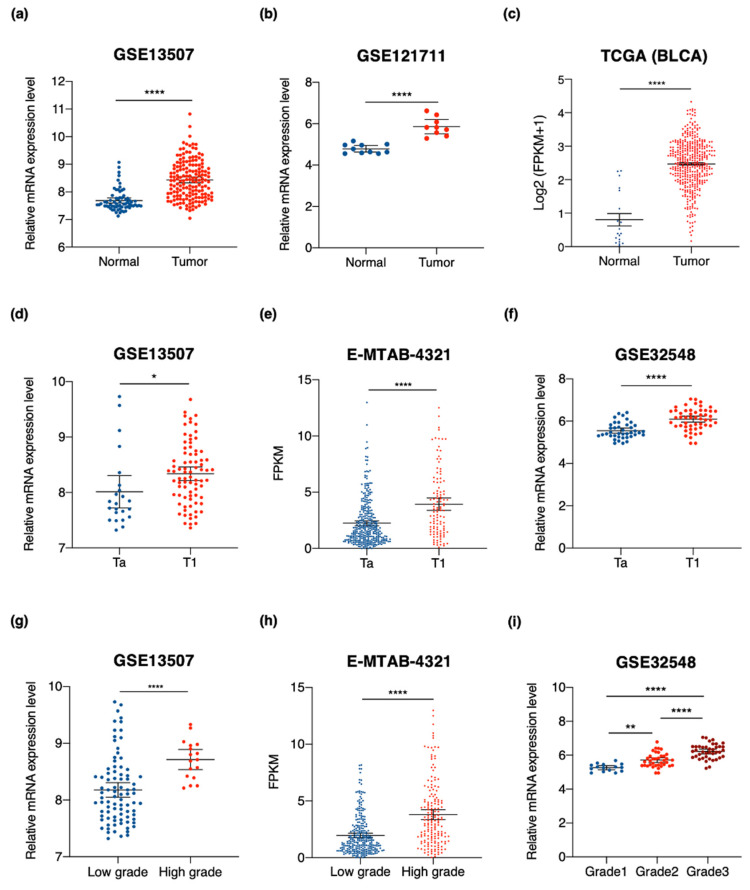
*SKA3* is upregulated in bladder tumors and associated with clinicopathological characteristics of patients with NMIBC. (**a**–**c**) Comparison of *SKA3* expression levels between normal bladder tissues and bladder cancer tissues. (**d**–**i**) *SKA3* expression levels downloaded from public datasets. *SKA3* expression levels in public datasets with tumor stage and grade. Data are means ± SEM. Unpaired samples were determined by Welch’s *t*-tests, respectively. * *p* < 0.05, ** *p* < 0.01, **** *p* < 0.0001.

We then analyzed independent cohorts from public data repositories to validate the prognostic potential of *SKA3* in NMIBC patients. For all three cohorts, *SKA3* was significantly upregulated in bladder tumors compared with normal bladder tissues. Thus, upregulation of *SKA3* is associated with bladder cancer development (Figure 4a–c). Tumor stage and grade reflect the biological aggressiveness of the disease and are important prognostic factors used to predict recurrence and progression in NMIBC patients [44]. In NMIBC tissues, *SKA3* overexpression was correlated with these clinical characteristics. *SKA3* expression increased with tumor stage (Figure 4d–f) and grade (Figure 4g–i). Overall, these results demonstrated that *SKA3* expression is promising as an accurate prognostic marker in NMIBC patients.

### 3.4. High SKA3 Expression Is Correlated with NMIBC Patient Prognosis

**Figure 5:**
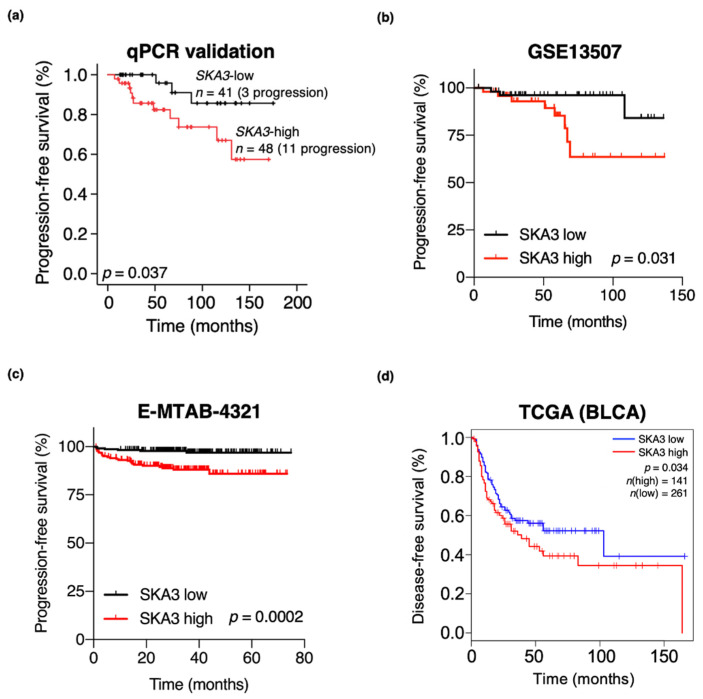
Kaplan–Meier survival plots showing effects of *SKA3* on poor clinical outcomes of patients with bladder cancer. (**a**) NMIBC patients were divided into lower 50th percentile (*n* = 41; three progressed) and upper 50th percentile (*n* = 48; 11 progressed) groups based on *SKA3* mRNA expression in tissues from NMIBC patients in validation cohort. PFS rates were significantly higher in low *SKA3* expression group than high *SKA3* expression group (log-rank test; *p* < 0.05). (**b**) NMIBC patients were divided into lower 50th percentile (*n* = 53; three progressed) and upper 50th percentile (*n* = 50; eight progressed) groups based on *SKA3* mRNA expression in tissues from NMIBC patients in GSE13507 cohort. PFS rates of NMIBC patients were significantly higher in low *SKA3* expression group than high *SKA3* expression group (log-rank test; *p* < 0.05). (**c**) NMIBC patients were divided into lower 50th percentile (*n* = 225; six progressed) and upper 50th percentile (*n* = 225; 25 progressed) groups based on *SKA3* mRNA expression in tissues from NMIBC patients in E-MTAB-4321 cohort. PFS rates were significantly higher in low *SKA3* expression group than high *SKA3* expression group (log-rank test; *p* < 0.001). (**d**) Kaplan–Meier survival curves and log-rank test of PFS in NMIBC patients with low vs. high SKA3 expression. SKA3 expression levels of *SKA3* high (*n* = 141) and *SKA3* low (*n* = 261) groups in TCGA cohort. DFS rates of NMIBC patients were significantly higher in low *SKA3* expression group than high *SKA3* expression group (log-rank test; *p* < 0.05). NMIBC, non-muscle invasive bladder cancer; PFS, progression-free survival; DFS, disease-free survival.

To evaluate the role of *SKA3* in predicting NMIBC patient prognosis, we conducted univariate and multivariate Cox regression analyses of the qPCR validation cohort with long-term follow-up. In NMIBC patients, *SKA3* expression was an independent predictor of PFS (HR, 4.155; 95% CI, 1.043–16.544; *p* = 0.043) (Table 3). To estimate whether *SKA3* expression has clinical implications, we classified NMIBC patients into *SKA3* high and *SKA3* low groups based on their median *SKA3* expression levels (Figure S1a–c). A Kaplan–Meier survival analysis showed that the NMIBC patients in the *SKA3* high-expression groups had significantly shorter PFS than those in the *SKA3* low-expression groups of the qPCR validation (log-rank test; *p* = 0.037; Figure 5a), GSE13507 (log-rank test; *p* = 0.031; Figure 5b), and E-MTAB-4321 (log-rank test; *p* = 0.0002; Figure 5c) cohorts. In the GSE13507 cohort, the *SKA3* high-expression group also had a significantly shorter RFS than the *SKA3* low-expression group (Figure S1d). In the TCGA cohort (log-rank test; *p* = 0.034; Figure 5d), the MIBC patients with high *SKA3* expression were correlated with worse prognoses than those with low *SKA3* expression. Several candidate genes were analyzed in multiple cohorts (Figure S2). These results suggest that *SKA3* is a promising prognostic marker for NMIBC patients.

### 3.5. SKA3 Expression Identified in an Epithelial Cell Subpopulation of Bladder Cancer

**Figure 6:**
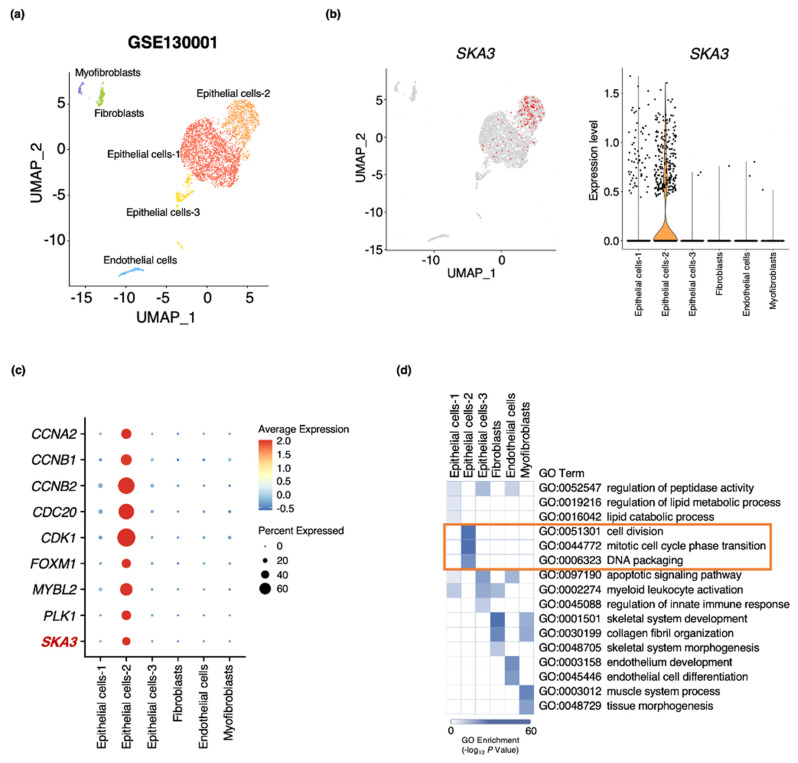
Single-cell transcriptome analysis of bladder cancer tissues. (**a**) UMAP plots of published scRNA-seq data for CD45-negative cells from bladder cancer tissues. (**b**) UMAP and violin plots displaying *SKA3* expression in all cell clusters in bladder cancer patients. (**c**) Dot plot showing expression of representative genes involved in latter cell cycle stages. (**d**) GO analysis of marker genes in bladder cancer cell clusters.

Bladder cancer is heterogeneous at the cellular and transcriptional levels [11]. To investigate *SKA3* expression in the various cellular compartments of bladder tumor tissues, we reanalyzed a public single-cell RNA sequencing (scRNA-seq) dataset performed on CD45-negative cells acquired from MIBC patients [41]. We categorized 3774 cells into six clusters based on gene expression patterns (Figure 6a and Figure S3a). Those in the Epithelial cells-2 cluster within the epithelial compartment showed the highest *SKA3* expression level (Figure 6b). According to the CBNUH RNA-seq data (Figure 1 and Figure 2), the cells in this cluster showed the highest expression levels of the genes implicated in the latter stages of the cell cycle including *CCNA2*, *CCNB1*, *CCNB2*, *CDC20*, *CDK1*, *FOXM1*, *MYBL2*, and *PLK1* (Figure 6c). A GO analysis of the six clusters furnished evidence that each subpopulation was correlated with unique biological functions in bladder cancer. For example, myofibroblasts and Epithelial cells-3 clusters were significantly associated with the muscle system and the apoptotic signaling pathway, respectively. The predicted functions of the Epithelial cells-2 cluster resembled those of the upregulated DEGs identified in the CBNUH RNA-seq dataset (Figure 6d). *MKI67* encoding a cell proliferation marker showed the highest expression level in the Epithelial cells-2 cluster (Figure S3b). These data indicate that the epithelial cell subpopulation strongly expressing *SKA3* may contribute to aggravating bladder cancer.

### 3.6. SKA3 Promotes Cell Proliferation and Cell Cycle Progression in Bladder Cancer

**Figure 7:**
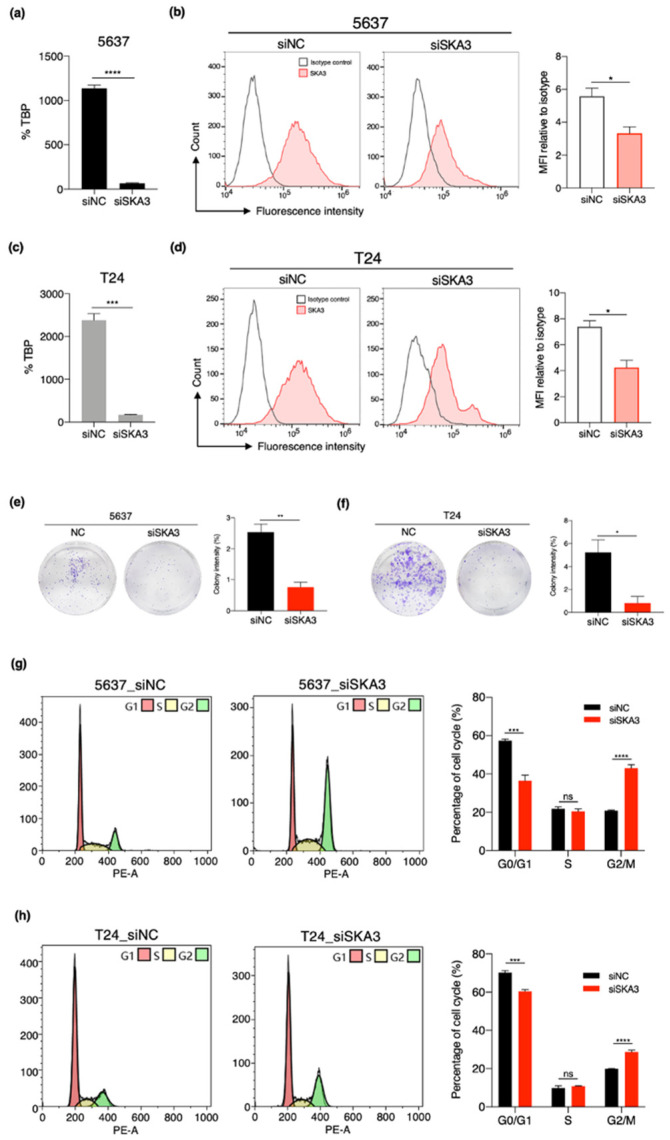
*SKA3* knockdown suppressed bladder cancer cell proliferation by inhibiting G2/M transition. (**a**,**b**) RT-qPCR results showing specific inhibition of *SKA3* expression by siRNA transfection. (**c**,**d**) Representative flow cytometry histograms of *SKA3* expression in 5647 and T24 cell lines treated with siNC or siSKA3 (left). SKA3 protein levels (MFI relative to isotype) in each cell line in triplicate (right). (**e**,**f**) Colony formation assay showing that *SKA3* promotes proliferation of bladder cancer cell lines 5637 and T24. (**g**,**h**) Cell cycle progression assay of 5637 and T24 cell lines transfected either with non-targeting control (NC) or *SKA3* siRNA. Cell cycle analysis data measured by flow cytometry. Error bar: means ± SEM. *P* values determined by Welch’s *t*-test. * *p* < 0.05, ** *p* < 0.01, *** *p* < 0.001, **** *p* < 0.0001. MFI, mean fluorescence intensity.

To determine the functional roles of *SKA3* in bladder cancer cells, we used small interference RNA (siRNA)-induced knockdown to suppress *SKA3* expression in the bladder cancer cell lines 5637 and T24. RT-qPCR showed that *SKA3* was significantly downregulated in bladder cancer cells transfected with *SKA3* siRNAs, and knockdown efficiency was approximately 90–95% (Figure 7a,b). Consistent with the *SKA3* mRNA levels, *SKA3* knockdown significantly reduced the SKA3 protein levels (Figure 7c,d). No other gene was affected by siRNA transfection targeting *SKA3* (Figure S4). We observed marked decreases in the proliferation of both bladder cancer cell lines in response to *SKA3* knockdown (Figure 7e,f). We analyzed the cell cycle by flow cytometry to elucidate the mechanisms by which *SKA3* regulates bladder cancer cell proliferation. A significant proportion of the *SKA3* siRNA-transfected bladder cancer cells had shifted to G2/M and fewer of them were in G0/G1 than the control (Figure 7g,h). Hence, *SKA3* knockdown inhibited G2/M transition. These results indicate that upregulation of *SKA3* may contribute to bladder cancer cell proliferation by controlling cell cycle progression.

## 4. Discussion

NMIBC is a clinically and molecularly heterogeneous disease. Even after patients with NMIBC are treated by transurethral resection of bladder tumor (TURBT) or intravesical therapy, ≤40–50% of them eventually progress to MIBC [44]. Moreover, NMIBC with a high risk of progression to MIBC is associated with poor prognosis [45]. Therefore, it is crucial to identify key biomarkers that can predict clinical outcomes in NMIBC.

We used a highly effective integrative transcriptome approach to detect sensitive, specific biomarkers for bladder cancer. Analysis of CBNUH RNA-seq data revealed that 1288 genes were differentially expressed between NMIBC and NAT. Most of the upregulated genes were associated with the cell cycle which was already known to be characteristic of bladder cancer. These results suggest that our dataset merited investigation and certain genes therein could serve as biomarkers. We used the upregulated DEGs in large independent bladder cancer patient cohorts to validate candidate genes with high detectability. A Cox regression analysis of an internal independent validation cohort with long-term f/u period and a public dataset used to build predictive models of time-event data revealed other genes that could predict NMIBC prognosis.

*SKA3* is a vital component of cell division as it blocks the spindle checkpoint [46]. However, it was unknown whether it could serve as a marker predicting the prognosis of bladder cancer patients. Here, *SKA3* expression was confirmed by RT-qPCR in NMIBC tissues to establish it as a novel biomarker in NMIBC patients. Moreover, Kaplan–Meier curves of time to progression in various external cohorts from public databases verified that *SKA3* is closely related to bladder cancer clinical pathology and progression. Thus, *SKA3* upregulation in NMIBC is strongly associated with disease progression. These results suggest that *SKA3* is an excellent prognostic marker to predict progression in NMIBC patients. High *SKA3* expression is also related to tumor progression in cervical, colorectal, liver, and pancreatic cancers [28,29,47,48,49]. The foregoing studies demonstrated that *SKA3* upregulation is clinically associated with poor cancer prognosis.

Our functional study showed that *SKA3* knockdown inhibited cancer cell colony formation and cell cycle progression from G2 to M. *SKA3* upregulation in the tumor microenvironment accelerates G2/M transition, promotes cell proliferation, and induces bladder cancer progression. Several studies indicated that *SKA3* regulates cell cycle transition and promotes cell proliferation in cervical, colorectal, and liver cancers [28,29,47]. Furthermore, scRNA-seq data revealed high *SKA3* and *MKI67* expression in specific epithelial cell subpopulations of bladder cancer tissue. These findings suggest that bladder cancer cell subpopulations with high *SKA3* expression are more likely to proliferate than others. Overall, *SKA3* may exacerbate bladder cancer tumors by affecting the cell cycle.

Cell cycle dysregulation typically occurs in cancer cells [50]. The most frequently investigated dysregulating factors in bladder cancer are the genes that control the cell cycle. Recent studies revealed that cell cycle gene expression patterns are characteristic of various NMIBC subtypes [43,51,52]. Hedegaard et al. found that most high-grade patients were grouped with those having the lowest PFS (Class 2) and that both high-risk NMIBC and MIBC had similar patterns [43]. Lindskrog et al. reported that NMIBC patients in the UROMOL 2021 Class 2a classification were at the highest risk of progression and had strongly upregulated late cell cycle genes [51]. Based on the UROMOL 2021 data, we confirmed that NMIBC patients in Class 2a had the highest *SKA3* expression levels. This manifestation was associated with low PFS in all patients with NMIBC as well as those in Class 2a (data not shown). A recent study demonstrated that the cell cycle gene *SKA3* is highly expressed in high-grade, non-muscle invasive tumors and is significantly related to disease progression.

A combination treatment consisting of intravesical immunotherapy with BCG is a standard protocol for NMIBC patients at high risk of progression [32,53]. Responsiveness to BCG immunotherapy is an important prognostic factor in predicting progression. Approximately 10–20% of all responders and 60–70% of all non-responders progress to MIBC [54,55,56]. In the future, functional studies should be conducted to determine whether a combination therapy comprising *SKA3* inhibition plus BCG can effectively prevent bladder cancer progression. Another goal is to clarify the mechanisms and effects of *SKA3* as a therapeutic target.

## 5. Conclusions

In the present study, we performed systematic transcriptome analyses on bladder cancer patient cohorts and identified *SKA3* as a putative accurate prognostic marker for progression. Subsequent functional studies on *SKA3* in bladder tumors may help detect other novel therapeutic targets as well.

## Figures and Tables

**Table 1:** Clinicopathological features of NMIBC patients and NAT examined in this study.

Characteristics	NMIBC	NAT	*p* Value
No.	16	8	
Mean age ± SD	60.5 ± 10.3	60.0 ± 11.5	0.809
Gender (%)			0.722
Male	13 (81.25%)	6 (75%)	
Female	3 (18.75%)	2 (25%)	
Grade, 2004 WHO grading system (%)			
Low	11 (68.75%)		
High	5 (31.25%)		
Stage (%)			
TaN0M0	11 (68.75%)		
T1N0M0	5 (31.25%)		

NMIBC, non-muscle invasive bladder cancer; NAT, normal adjacent tissue; SD, standard deviation. *p* value obtained using the Student *t*-test or Chi-squared test.

**Table 2:** Clinicopathological features of primary NMIBC patients and control tissues (normal bladder mucosae).

Variable	NMIBC	Control	*p* Value
No.	89	16	
Mean age ± SD	68.00 ± 13.68	68.00 ± 15.63	0.879
Gender (%)			0.104
Male	76 (85.4%)	16(100%)	
Female	13 (14.6%)		
Tumor size (%)			
≤1 cm	51 (57.3%)		
2–3 cm	38 (42.7%)		
>3 cm	0		
Multiplicity (%)			
Single	50 (56.2%)		
2–7	26 (29.2%)		
>7	13 (14.6)		
Grade, 2004 WHO grading system (%)			
Low	65 (73.0%)		
High	24 (27.0%)		
Stage (%)			
TaN0M0	26 (29.2%)		
T1N0M0	63 (70.8%)		
BCG therapy (%)			
No	57 (64.0%)		
Yes	32 (36.0%)		
Recurrence—no. of patients (%)			
No	54 (60.7%)		
Yes	35 (39.3%)		
Progression—no. of patients (%)			
No	75 (84.3%)		
Yes	14 (15.7%)		
Survival—no. of patients (%)			
Alive	60 (67.4%)		
Death	29 (32.6%)		
Mean follow-up (range)—months	70.12 (10.70–174.90)		

BCG, Bacillus Calmette-Guerin; NMIBC, non-muscle invasive bladder cancer; SD, standard deviation. *p* value obtained using Kruskal–Wallis H test (NMIBC compared with control).

**Table 3:** Univariate and multivariate Cox regression analyses to predict NMIBC progression.

Variables	Univariate Cox Analysis		Multivariate Cox Analysis	
	HR (95% CI)	*p* Value	HR (95% CI)	*p* Value
Age	1.086 (1.020–1.156)	0.009 *	1.083 (1.014–1.157)	0.017 *
Gender			0.104	
Male (Ref.) vs. Female	1.311 (0.290–5.932)	0.725		
Tumor size				
≤1 cm (Ref.) vs. 2–3 cm	1.310 (0.378–4.536)	0.670		
Multiplicity				
Single	Ref.	0.106		
2–7	2.176 (0.531–8.910)	0.280		
>7	7.381 (1.153–47.252)	0.035 *		
2004 WHO Grade				
Low (Ref.) vs. High	5.132 (1.757–14.987)	0.003 *	1.926 (0.580–6.390)	0.284
Stage				
Ta (Ref.) vs. T1	0.698 (0.233–2.087)	0.520		
BCG				
No (Ref.) vs. Yes	1.663 (0.575–4.808)	0.348		
SKA3 expression				
High expression (Ref.) vs. Low expression	3.413 (1.065–10.930)	0.039 *	4.155 (1.043–16.544)	0.043 *

BCG, Bacillus Calmette-Guerin; CI, confidence interval; HR, hazard ratio; Ref., reference. * *p* < 0.05.

## Data Availability

All publicly available datasets used in this study were described in the Materials and Methods section. Other datasets generated in this study are available upon reasonable request to the corresponding author.

## Supplementary Materials

The following are available online at https://www.mdpi.com/article/10.3390/cancers13184673/s1, Figure S1: SKA3 predicts poor clinical outcome; Figure S2: Survival analysis for the expression of candidate genes in patients with bladder cancer; Figure S3: Analysis of public single-cell RNA sequencing data of bladder cancer; Figure S4: Knockdown of SKA3 in bladder cancer cell lines by siRNA transfection.
