# Supplementary material for: Integrative Transcriptome Profiling Reveals SKA3 as a Novel Prognostic Marker in Non-Muscle Invasive Bladder Cancer
Source: Cancers (Basel). 2021 Sep 17;13(18):4673. doi: 10.3390/cancers13184673 (PMC8470398; doi:10.3390/cancers13184673)
Supplement: Supplementary file 1 [file cancers-13-04673-s001.zip › cancers-1320924-supplementary.pdf]

Supplementary Materials

Integrative Transcriptome Profiling Reveals Prognostic Marker in Non-Muscle Invasive Bladder Cancer

Chaelin You, Xuan-Mei Piao, Keunsoo Kang, Yong-June Kim and Kyuho Kang

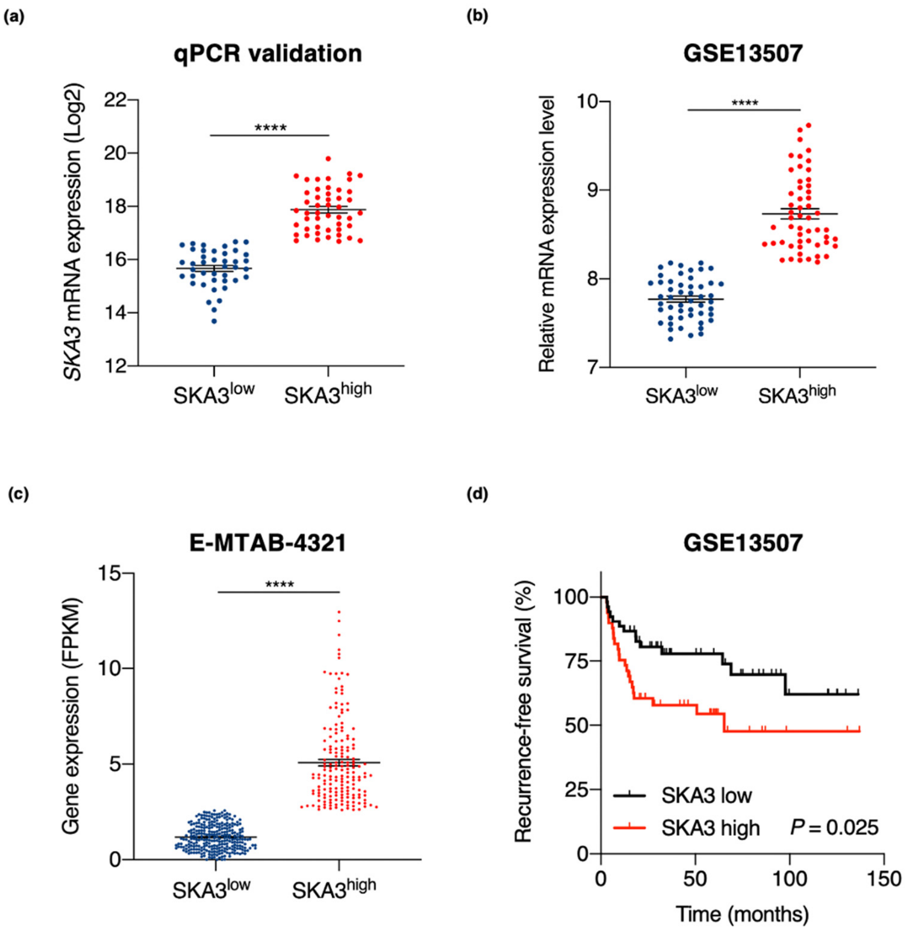

Figure S1. SKA3 predicts poor clinical outcome. (**a**) SKA3 expression levels of SKA3 high (n=48) and SKA3 low (n=41) groups in qPCR validation cohort; (**b**) SKA3 expression levels of SKA3 high (n=50) and SKA3 low (n=53) groups in GSE13507 cohort; (**c**) SKA3 expression levels of SKA3 high (n=225) and SKA3 low (n=225) groups in E-MTAB-4321 cohort; (**d**) NMIBC Patients were divided into two groups, lower 50th percentile (n=50, 14 of them recurred) and upper 50th percentile groups (n=53, 22 of them recurred), according to the mRNA expression level of SKA3 in GSE13507 cohort. The recurrence-free survival rate of NMIBC patients was significantly higher in the low SKA3 expression group (log-rank test, $p < 0.05$). NMIBC, non-muscle invasive bladder cancer; RFS, recurrence-free survival. Error bar: mean value with SEM. p values were determined by Welch's t-test. *$p < 0.05$, **$p < 0.01$, ***$p < 0.001$, ****$p < 0.0001$.

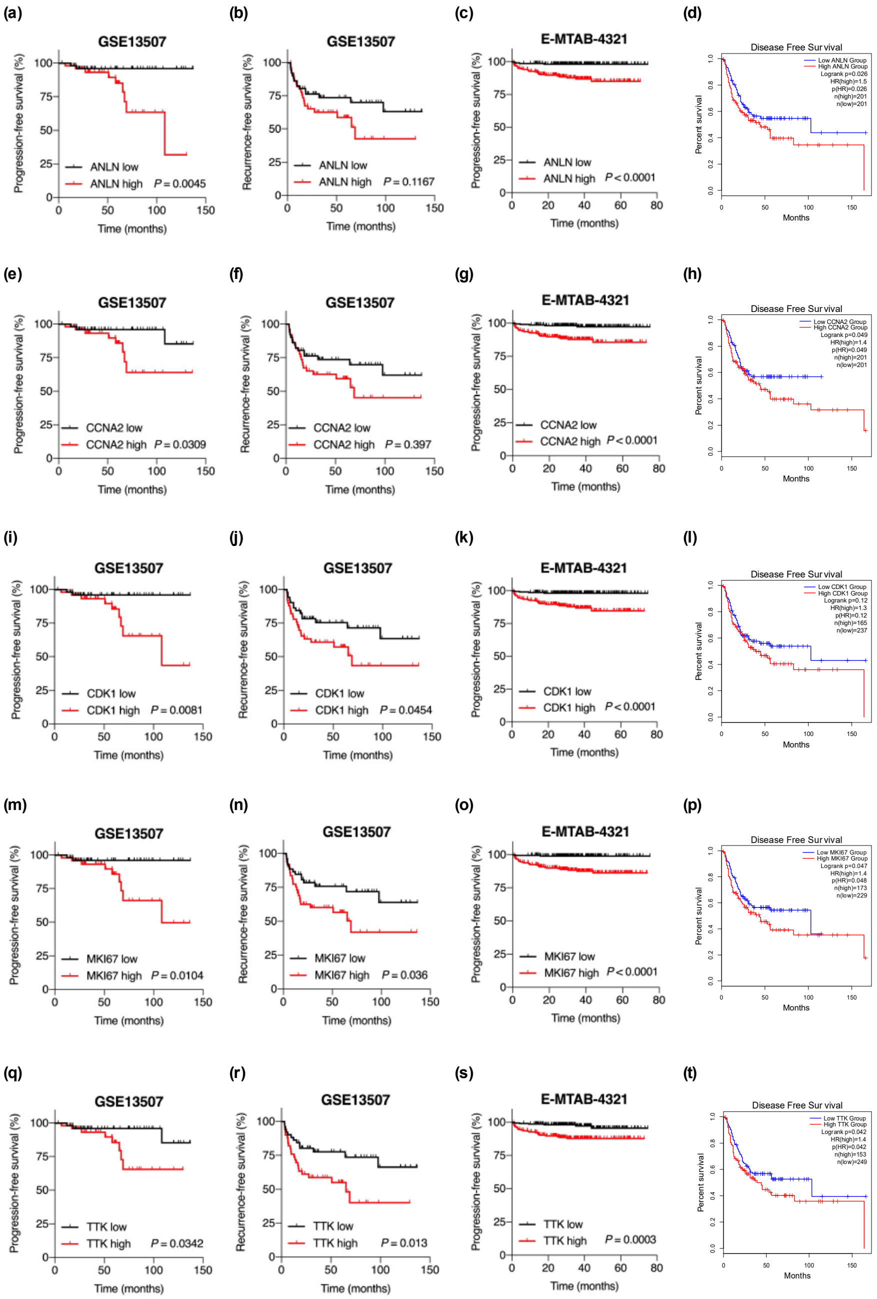

Figure S2. Survival analysis for the expression of candidate genes in patients with bladder cancer. **(a–d)** Kaplan-Meier survival analysis in two groups according to the mRNA expression level of ANLN in multiple cohorts; (**e–h**) Kaplan-Meier survival analysis in two groups according to the mRNA expression level of CCNA2 in multiple cohorts; (**i–l**) Kaplan-Meier survival analysis in two groups according to the mRNA expression level of CDK1 in multiple cohorts; (**m–p**) Kaplan-Meier survival analysis in two groups according to the mRNA expression level of MKI67 in multiple cohorts; (**q–t**) Kaplan-Meier survival analysis in two groups according to the mRNA expression level of TTK in multiple cohorts.

(a)

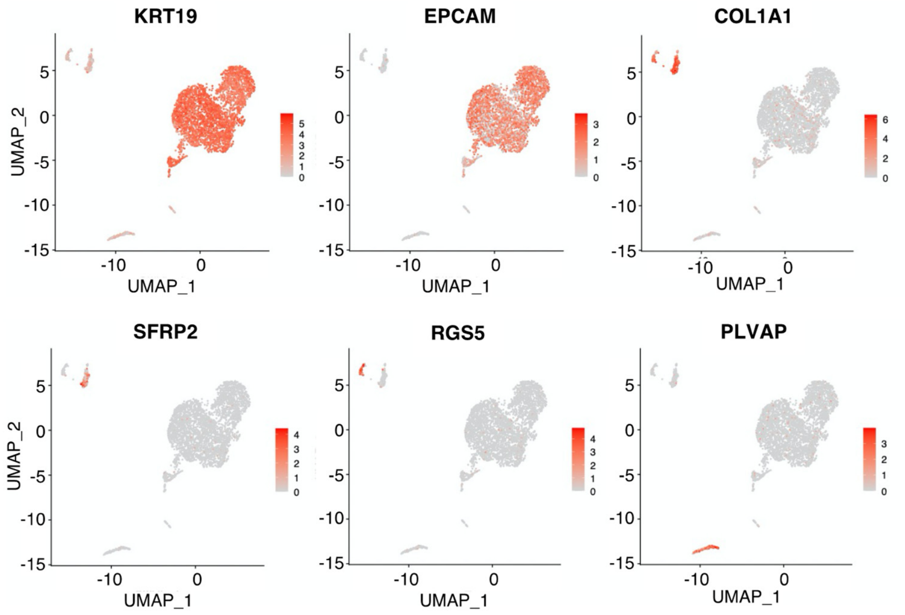

(b)

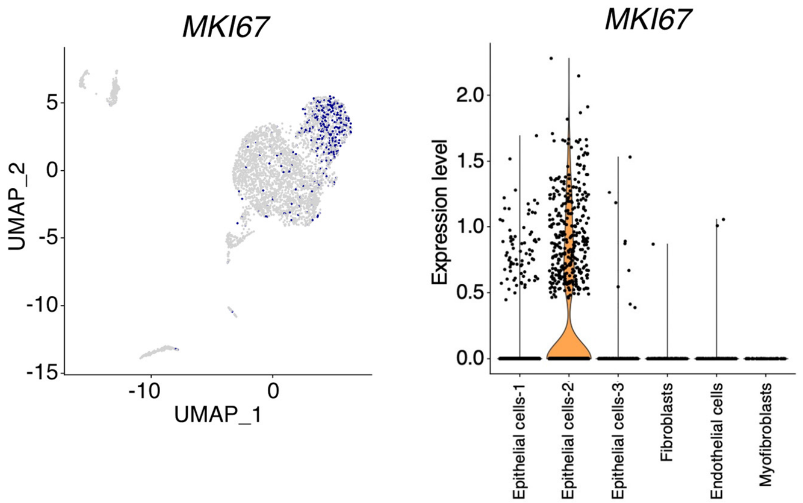

Figure S3. Analysis of public single-cell RNA sequencing data of bladder cancer. (**a**) Feature plots of the characteristic markers for all cell types and their expression levels analyzed by Seurat; (**b**) UMAP plot and violin plot displaying the expression of MKI67 in all cell clusters in bladder cancer patients.

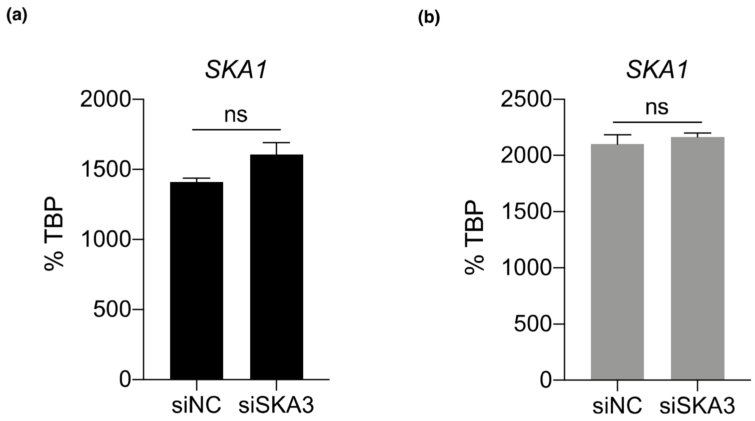

Figure S4. Knockdown of SKA3 in bladder cancer cell lines by siRNA transfection. SKA1 expression levels were analyzed by RT-qPCR in 5637 cell line (**a**) and T24 cell line (**b**). Error bar: mean value with SEM. *p* values were determined by Welch's *t*-test.
